# Supplemental Nutrition Assistance Program issuance timing is associated with sugar-sweetened beverage marketing in the USA

**DOI:** 10.1017/S1368980024001563

**Published:** 2024-09-23

**Authors:** Jane Dai, Erica L Kenney, Mark J Soto, Anthony Zhong, Alyssa J Moran, Emily M Broad Leib, Sara N Bleich

**Affiliations:** 1 Department of Health Systems and Population Health, University of Washington, Seattle, WA, USA; 2 Department of Health Policy and Management, Harvard TH Chan School of Public Health, Boston, USA; 3 Department of Nutrition, Harvard TH Chan School of Public Health, Boston, USA; 4 Department of Health Policy and Management, Johns Hopkins Bloomberg School of Public Health, Baltimore, USA; 5 Harvard Law School, Cambridge, USA

**Keywords:** Beverage marketing, SNAP, Nutrition policy, Retail stores

## Abstract

**Objective:**

Prior research has shown that there are more supermarket displays of sugar-sweetened beverages (SSB) during times when Supplemental Nutrition Assistance Program (SNAP) benefits are distributed (‘issuance periods’). This may contribute to inequitable purchasing and consumption. This study examines whether SSB marketing in weekly supermarket circulars, which retailers use to advertise products, is more prevalent during issuance periods compared to non-issuance periods.

**Design:**

We conducted longitudinal, difference-in-differences analyses of data extracted from weekly supermarket circulars of randomly selected SNAP-authorised retailers in six states. Analyses tested whether SSB advertisements (‘ads’) were more prevalent during SNAP issuance periods compared to non-issuance periods within states with distinct issuance periods (3, 5, 10 or 15 d), compared to one state with continuous benefit issuance (28 d; the ‘control’ state).

**Setting:**

Weekly online supermarket circulars collected from August to September 2019 were analysed in 2021.

**Participants:**

The study sample included 5152 circulars from 563 SNAP-authorised retailers in the states California, Connecticut, Nebraska, New Jersey and Texas (distinct issuance period states) as well as Florida (‘control’ state).

**Results:**

The estimated mean percentage of beverage ads classified as SSB ads during issuance days was 51·5 % compared to 48·4 % during non-issuance days (*P* < 0·001). In difference-in-differences analyses comparing to the ‘control’ state with continuous issuance, SSB ad counts were 2·9 % higher (95 % CI 1·9 %, 3·9 %) during SNAP issuance relative to non-issuance.

**Conclusions:**

SSB ads are slightly more prevalent in weekly supermarket circulars during SNAP issuance periods. Future research should explore the linkages between circular ads and SSB purchasing and consumption.

In the USA and globally, consumption of sugar-sweetened beverages (SSB), which increases the risk of developing type 2 diabetes mellitus and other chronic diseases, remains high^(1–3)^. Despite gradual declines in SSB consumption over the past two decades in the USA, disparities in SSB consumption persistently reflect socio-economic gradients, such that those with lower incomes tend to consume higher amounts of this harmful product^(4,5)^.

In fiscal year 2022, Supplemental Nutrition Assistance Program (SNAP) helped more than 41 million US adults and children in low-income households purchase food each month^(6)^, distributing cash-like benefits to eligible recipients that can be used to purchase food from more than 240 000 authorised retailers^(7)^. Strong evidence suggests that SNAP helps to lift families out of poverty^(8)^, but the impact of SNAP on diet quality is mixed. For example, although SSB consumption and purchasing is common across all American households regardless of SNAP participation^(9,10)^, some evidence suggests that SNAP participants purchase and consume more SSB than higher-income and SNAP-eligible non-participants^(11,12)^.

One potential driver of these disparities in SSB intake by SNAP participation may be differential exposure to SSB product promotions. The grocery store industry spent $1·8 billion on advertising in 2021^(13)^, and evidence suggests that people are more likely to purchase advertised foods and beverages^(14,15)^. For food retail stores, marketing is curated by food and beverage retailers through promotion practices, including price discounts and product recommendations^(14,16)^. These promotion practices impact how products are priced, placed and promoted – which can then reach consumers through advertisements they may receive in the mail or digitally (e.g. circulars) or in stores themselves^(14)^. For example, within the in-store context, shoppers browsing the aisles can view curated selections of SSB products and make their purchasing decisions based on how well their decision-making criteria (e.g. affordability, quality and desirability) align with the retailer’s decisions on what, where and how products are stocked^(17)^. Research has demonstrated consistently that product sales are significantly influenced by in-store marketing such as end-of-aisle promotional displays^(18–20)^ and reduced prices^(21,22)^.

However, exposure to SSB marketing is not constrained to the in-store setting, as individuals often receive mail and digital circulars prior to physically stepping inside a grocery store^(23)^. Through weekly circulars, selected in-store promotions – such as end-of-aisle promotional displays and price reductions – can be directly advertised to local households. Circulars are distributed either through print mailers or online, the latter of which is done through posting directly on the retailer website or through retailer-specific digital platforms (e.g. Target’s mobile application). The most appealing in-store promotions may then be purposely selected to be put on display in circulars, potentially nudging shoppers to deviate from a healthier grocery list^(24,25)^ and purchase items they had not originally considered^(15,26)^. Today, most circulars are distributed digitally^(23,27)^. Although SNAP participants may have less Internet access than SNAP non-participants, the majority of SNAP participants do have access to the Internet^(28,29)^. Thus, digital supermarket circulars are still relevant to how SNAP participants plan their grocery shopping.

Increased exposure to SSB marketing may also coincide with the timing of when SNAP participants receive their benefits, as SNAP-authorised retailers may be incentivised to adjust their SSB marketing strategies to align with the timing of when SNAP-participating households receive and spend their benefits. Most SNAP benefits are spent within the first week of receipt^(30)^, and emerging evidence suggests that increases in SSB marketing coincide with SNAP benefit issuance periods^(31,32)^. However, benefit issuance periods can also vary, as individual states have the authority to determine when benefits from this federal programme are distributed each month. In some states, benefits are distributed to all households on the same day of the month (e.g. all households receive benefits on the first day of the month); in other states, benefits are distributed over a longer period, with households receiving benefits on different days depending on their last name or case number (e.g. one household receives benefits on the fifth day of each month and another receives benefits on the twenty-third day of each month)^(33)^. To our knowledge, only one cross-sectional study conducted in New York has examined associations between SNAP benefit issuance and beverage marketing, finding that in-store SSB marketing was positively associated with the period of time when SNAP benefits are issued^(31)^.

This study builds on existing literature by examining the associations between SNAP benefit timing and beverage marketing across multiple states using a longitudinal quasi-experimental design that estimates whether there are increases in SSB marketing during SNAP benefit issuance times in states with distinct issuance periods, controlling for potential confounding by cyclical time trends by comparing any changes in these states with patterns of SSB marketing in a state with no distinct benefit issuance period. Instead of studying in-store marketing practices, we leverage novel longitudinal data from weekly supermarket circulars, an influential type of digital and print marketing with weekly sales promotions^(34)^. Circulars can be considered a type of ‘cooperative advertising’ and are perhaps one of the few out-of-store promotion practices that effectively increase sales^(14,27)^. In fact, despite today’s digital era of grocery retail, circulars remain as the most successful promotion practice that grocery retailers have at their disposal: up to 85 % of consumers browse circulars to identify deals, and half these consumers review circulars from multiple stores to decide where to grocery shop^(23,35,36)^. We hypothesised that the prevalence of SSB advertisements in weekly circulars would be higher during benefit issuance periods compared to non-issuance periods.

## Methods

### Study design

In this study, we used a natural experiment design with a difference-in-differences approach to test whether states with distinct SNAP benefit issuance periods (short, long) saw higher levels of SSB advertising in weekly grocery circulars during in-issuance periods compared to during non-issuance periods, compared to a state with no distinct SNAP benefit issuance period (continuous).

### Study sample

We collected weekly circulars over 8 weeks (August to September 2019) from a sample of SNAP-authorised retailers from six states – California (CA), Connecticut (CT), Florida (FL), Nebraska (NE), New Jersey (NJ) and Texas (TX). We determined this study period as appropriate for examining marketing in circulars, as most other 2-month sequential periods in the calendar year overlapped with national and religious holidays that could lead to atypical trends in the types of beverages that are promoted.

To select the states for this study, we used administrative data from the United States Department of Agriculture (USDA) to stratify all states by SNAP issuance period. We first grouped the states into one of two categories: short issuance (all households receive SNAP benefits within the first 7 d of the month) or extended issuance (households receive SNAP benefits past the first 7 d of the month)^(33)^. We then selected the three states with the highest annual SNAP redemption revenue in each issuance group^(37)^ that also had at least 100 retailers meeting our inclusion criteria as described below (six states total). CT (3 d), NE (5 d) and NJ (5 d) were selected from the list of short-issuance states; CA (10 d), TX (15 d) and FL (28 d) were selected from the list of extended issuance states. We then further stratified extended issuance into two categories of long issuance (CA and TX) and continuous issuance (FL). For the purposes of analyses in this study, we selected Florida as the comparison state as it has a nearly month-long (continuous) issuance period, as opposed to the distinct issuance period (during specific weeks) found in the other states. With no specific time period each month for issuance of SNAP benefits in Florida, there would be no incentive for grocery retailers in Florida to use marketing strategies related to capitalising on the monthly SNAP benefit redemption cycle that could be present in states with shorter issuance periods.

Within each selected state, we identified SNAP-authorised retailers through the SNAP retail locator from the USDA in July 2019^(38)^. We classified retailers by large (e.g. supermarkets and superstores) and small (e.g. convenience and pharmacy) store status using an existing framework^(7)^. We then randomly sampled eighty-two large stores and eighteen small stores within each state to match the distribution of where national SNAP dollars are spent in retail contexts^(7)^ for a target sample population of 600 stores (492 large and 108 small). Stores were eligible for the study if they had a weekly circular available online and were open for business; we sampled an additional twelve large stores and eight small stores in case retailers became ineligible (e.g. closure during the study). Stores with zero beverage ads in any circular were excluded (*n* 33 store locations, 311 circulars). We downloaded digital versions of each weekly circular from all retailers from August through September 2019. Each weekly circular contributed seven circular-day observations, and each circular-day was categorised based on whether it fell within the SNAP issuance period for that state in that calendar month.

Based on pilot data extraction, a Cohen’s *h* effect size of 0·1 with 80 % power required a minimum sample of 4030 circulars to test the difference in the ratio of SSB ads to all beverage ads comparing issuance to non-issuance at *P* < 0·05. Our final analytic sample included 5152 circulars across 563 stores (456 large and 107 small).

### Measures

For each circular in our sample, we recorded the total number of ads and categorised each ad as one of two types: beverage or other (e.g. food and household products). Ads were defined as an image or description of a product, or a cluster of products with a corresponding promotion, which we describe in more detail below.

Beverage ads were coded into four beverage categories: SSB; milk or 100 % juice; diet or low-calorie drinks; and unsweetened drinks. This classification strategy was adapted from previously published studies on food and beverage marketing^(39,40)^. See online Supplementary Information for our data extraction protocol. Ads typically promoted individual items; however, when multiple items were pictured in one ad (e.g. a ‘4 for $4’ ad of a SSB product alongside images of a low-calorie version and a SSB from another brand), we only coded for the most visually salient beverage, which we defined as either the largest pictured beverage or the first to be seen when reading (left to right, top to bottom).

To obtain demographic characteristics of the included retailers, we matched retailers’ addresses to US Census tracts and used the American Community Survey (2015–2019) to obtain estimates of the total population of the census tract, the proportion of households enrolled in SNAP, the proportion of households whose income was below the federal poverty level and the proportion of the population that was non-Hispanic White, non-Hispanic Black, non-Hispanic Other Race and Hispanic.

### Analysis

#### Descriptive analyses

For each state in the sample, we calculated descriptive statistics, including the means and standard deviations of the number of stores, the number of circulars, demographic characteristics of store location census tracts and circular characteristics. All states with distinct issuance periods (short and long) were then compared on these measures to Florida, the comparison state for this study based on its continuous issuance status, using *t* tests.

To first assess whether the frequency of SSB ads, as well as other beverage types, varied by SNAP benefit issuance period within each state, we used generalised linear regression models with a Poisson distribution and log-link function that tested the association of beverage ad counts that were SSB, diet drinks, milk/100 % juice or unsweetened drinks during benefit issuance periods compared to non-issuance periods. The models accounted for clustering of circular-weeks within stores and adjusted for state-level fixed effects. Florida, with its issuance period of 28 d, was excluded from this analysis because there was no non-issuance period for comparison.

#### Difference-in-difference analysis

Then, to more robustly evaluate whether SSB ads were more common during SNAP benefit issuance periods, and to adjust for potential time trends (i.e. SSB ads are more common at the beginning of a month regardless of SNAP benefit issuance), we used nested difference-in-differences generalised linear regression models with a Poisson distribution and log-link function to model the association between SNAP benefit issuance periods and counts of SSB ads compared to a control state with no distinct benefit issuance period due to it encompassing the full month (Florida). In each analysis where Florida is compared to another state, the circular-days in Florida are mapped to the longest issuance period of the comparison states and then each circular-day is categorised as in or out of issuance as described above. For example, when comparing California and Florida, the first 10 d of each month are considered ‘in issuance’ for circulars from Florida. Circular-weeks were nested within store locations to account for repeated observations of circular-days within circular-weeks and between-store differences in ad counts. Each model estimated the ratio of SSB ad appearances between the comparison states and Florida on days coinciding with each state’s SNAP issuance period relative to non-issuance days.

All models adjusted for the total number of all beverage ads and used robust standard errors and store-level random effects to account for variation in storewide attributes (e.g. demographic composition of census tracts)^(41)^. Subgroup analyses were conducted by length of benefit issuance period (short or long). Statistical significance was considered at *P* > 0·05. All analyses were conducted in Stata, version 15.

## Results

The mean number of ads per circular ranged from 122·4 (CA) to 300·0 (NJ), which on average, contained a range of 12·2 (NE) to 34·4 (NJ) beverage ads per circular (Table 1). Among beverage subtypes, the mean ads per circular ranged from 6·1 (NE) to 15·9 (NJ) for SSB, 1·7 (TX) to 6·4 (NJ) for milk or 100 % juice, 2·9 (NE) to 10·3 (NJ) for unsweetened beverages, and 1·0 (NE) to 1·8 (NJ) for diet and low-calorie beverages.

**Table 1:** Characteristics of grocery stores participating in Supplemental Nutrition Assistance Program (SNAP) (*n* 563) and weekly circulars (*n* 5152) across six states by issuance period for SNAP benefits, August to September 2019

	Short issuance^*^						Long issuance^†^				Continuous issuance^‡^	
	CT (3 d)		NE (5 d)		NJ (5 d)		CA (10 d)		TX (15 d)		FL (28 d)	
	Mean	sd	Mean	sd	Mean	sd	Mean	sd	Mean	sd	Mean	sd
Total # of store chains	20		27		11		30		15		9	
Total # of store locations	89		96		92		98		91		97	
Total # of circular-weeks^§^	823		854		903		888		811		873	
# locations per chain, mean (sd)	3·5	6·1	2·3	2·6	1·3	0·9	3·3	2·9	4·6	5·3	8·2	15·8
# circular-weeks per location, mean (sd)	**9·2**	**0·5**	8·9	0·6	**9·8**	**0·5**	9·1	0·4	8·9	0·4	9·0	0·4
% of stores that are ‘Large’	0·8	0·4	0·8	0·4	0·8	0·4	0·8	0·4	0·8	0·4	0·80·	0·40
Store level												
% White, mean (sd)	**70·6**	**22·7**	**79·5**	**15·2**	56·7	28·4	**42·5**	**24·3**	**48·8**	**25·5**	57·4	26·2
% Black, mean (sd)	**7·4**	**9·1**	**4·1**	**7·9**	10·9	15·1	**5·9**	**10·1**	11·9	14·5	12·8	16·9
% Other, mean (sd)	2·6	2·2	2·2	1·7	2·1	1·8	**3·3**	**2·3**	**1·8**	**1·5**	2·2	1·7
% Hispanic, mean (sd)	**13·5**	**15·8**	**11·0**	**12·6**	19·4	17·9	**36·7**	**24·1**	**32·7**	**25·1**	24·6	22·9
Total population of census tract, mean (sd)	**4710·1**	**1568·7**	**3972·7**	**1404·1**	5353·3	2121·6	5407·9	1953·5	7004·0	4696·5	6159·9	5114·9
% Households in SNAP, mean (sd)	10·5	9·9	**9·1**	**7·0**	**8·8**	**9·9**	9·9	8·7	11·8	9·7	12·4	9·5
% Households below poverty level, mean (sd)	**5·5**	**6·5**	8·4	7·2	8·0	9·3	**11·2**	**10·0**	10·7	8·2	8·9	6·2
Circular level												
# of all promotions per circular, mean (sd)	**246·6**	**140·2**	**133·8**	**53·1**	**300·0**	**168·9**	**122·4**	**64·6**	**128·9**	**43·8**	181·6	61·1
# of beverage promotions per circular, mean (sd)	**26·1**	**15·2**	**12·3**	**6·0**	**34·4**	**19·4**	**15·2**	**8·3**	**15·0**	**5·7**	19·8	6·6
# SSB promotions per circular, mean (sd)	**12·1**	**7·7**	**6·1**	**3·2**	**15·9**	**9·6**	**7·2**	**4·8**	**7·9**	**3·4**	9·6	4·1
# Milk/juice promotions per circular, mean (sd)	**4·8**	**3·8**	**2·3**	**2·1**	**6·4**	**4·5**	**2·4**	**2·1**	**1·7**	**1·5**	3·1	1·9
# Diet/low-calorie beverage promotions per circular, mean (sd)	**1·3**	**1·4**	1·0	1·2	**1·8**	**1·6**	**1·2**	**1·3**	**1·5**	**1·7**	1·0	1·1
# Unsweetened beverage promotions per circular, mean (sd)	**7·9**	**4·4**	**2·9**	**2·1**	**10·2**	**5·8**	**4·3**	**2·8**	**3·9**	**2·2**	6·1	2·2
# Mixed promotions per circular, mean (sd)^||^	**1·1**	**2·1**	**0·9**	**2·0**	1·9	2·5	1·9	3·0	**2·4**	**2·5**	2·1	1·8
# Circular-days in issuance, mean (sd)^¶^	**0·9**	**1·3**	**1·3**	**1·8**	**1·4**	**1·9**	**2·4**	**2·7**	**3·5**	**2·8**	6·4	1·1

SSB, sugar-sweetened beverages.

Authors analysed weekly circulars from 563 stores in six states collected from August to September 2019. Demographic characteristics are from ACS 2015 to 2019, 5-year estimates. **Bold** indicates statistically significant difference from Florida (FL) at *P* < 0·05.

* SNAP benefits are issued within the first 3 d of the month in CT and in the first 5 d in both NE and NJ, with distribution based on a number randomly assigned to beneficiaries.

† SNAP benefits are issued in the first 10 d of the month in CA based on the last digit of the participant’s social security number and in the first 15 d of the month in TX based on a number randomly assigned to beneficiaries.

‡ SNAP benefits are issued in the first 28 d of the month in FL based on a number randomly assigned to beneficiaries.

§ Circular-weeks are the 7 calendar days during which the circular and its promotions are in effect.

|| Mixed promotions include those with both beverage and non-beverage items, such as ‘5 items for $5’.

¶ Circular-day is defined as a single calendar day within a 7-d circular-week, and it is considered ‘in issuance’ if the circular-day coincides with a calendar day when SNAP benefits are issued in that state.

On average across the six states with distinct issuance periods, the estimated percentage of SSB ads among all beverage ads during issuance days was 51·5 % compared to 48·4 % during non-issuance days (Table 2). The estimated SSB ad counts were 1·1 % (95 % CI 0·06 %, 1·5 %) higher during SNAP issuance relative to non-issuance, or an additional 0·11 SSB ads per circular, while estimated diet beverage ad counts were 1·4 % higher (95 % CI 0·06 %, 2·2 %) during issuance, or an increase of 0·03 ads per circular. In contrast, the percent of milk or 100 % juice ads were 0·7 % lower (95 % CI 0·01 %, 1·3 %) during SNAP issuance periods compared to non-issuance, or a decrease of 0·03 ads per circular. There was no statistically significant change in water ads.

**Table 2:** Poisson regression models testing whether the proportions of ads promoting sugar-sweetened beverages out of all beverage ads differ by SNAP issuance period (30 117 advertising days across five states)

	Sugar-sweetened beverage ads		*P*-value	Diet or low-calorie beverage ads		*P*-value	Milk or 100 % juice ads		*P*-value	Water ads		*P*-value
	Mean	sd		Mean	sd		Mean	sd		Mean	sd	
Percentage of all beverage ads in circulars during SNAP issuance days, mean (sd)^*^	51·5	17·5		8·1	8·1		13·7	10·6		26·9	12·2	
Percentage of all beverage ads in circulars during non-SNAP issuance days, mean (sd)^†^	48·4	16·7		6·8	8·1		17·1	13·4		27·8	12·3	
Incident-rate ratio of circulars containing specific beverage ads during SNAP issuance days^‡^	1·010		**< 0·001**	1·015		**< 0·001**	0·993		**0·021**	0·998		0·543
95 % CI	1·006, 1·015			1·006, 1·023			0·987, 0·999			0·991, 1·005		
Absolute difference in number of specific beverage ads in circulars during SNAP issuance days^§^	0·107		**< 0·001**	0·030		**< 0·001**	−0·028		**0·021**	−0·012		0·543
95 % CI	0·060, 0·154			0·013, 0·047			−0·051, –0·005			−0·052, 0·028		
Observations	30 177			30 177			30 177			30 177		

SNAP, Supplemental Nutrition Assistance Program.

Authors’ analysis of daily ads derived from the weekly circulars of 470 stores in five states collected from August to September 2019. Sample excludes all 100 stores from Florida. Estimates are derived from Poisson regression models as incident-rate ratios and indicate the multiplicative effect of a circular-day coinciding with a SNAP issuance day on the ad counts in that circular-day. All models include state-level fixed effects, store location- and circular-level random effects, and robust standard errors that are clustered at the store level. Total number of beverage ads are included in each regression as an exposure with their coefficient constrained to 1.

Boldface indicates statistical significance at *P* < 0·05.

* Circular-days are considered ‘in issuance’ if the calendar day covered by a circular-week coincides with a day when SNAP benefits are issued in that state.

† Circular-days are considered ‘out of issuance’ if the calendar day covered by a circular-week coincides with a day when SNAP benefits are not issued in that state.

‡ Estimates represent the multiplicative effect of SNAP issuance on the total number of beverage promotions.

§ Estimates represent the absolute effect of SNAP issuance on the total number of beverage promotions obtained from the margins command in Stata, version 15.

In the difference-in-differences analyses comparing the states with distinct SNAP benefit issuance periods with Florida to account for possible non-SNAP-related time trends, we found that SNAP issuance was associated with an increase of 0·28 (95 % CI 0·19, 0·38) SSB ads, a 2·9 % increase (95 % CI 1·9 %, 3·9 %) on days during SNAP issuance relative to Florida (Table 3). Short-issuance states (CT, NE and NJ) had circulars with 0·34 (95 % CI 0·24, 0·44) additional SSB ads, a 3·1 % increase (95 % CI 2·2 %, 4·0 %) during SNAP issuance relative to Florida. Long-issuance states (CA and TX) had circulars with 0·21 (95 % CI 0·10, 0·32) additional SSB ads, a 2·5 % increase (95 % CI 1·1 %, 3·9 %) during SNAP issuance relative to Florida.

**Table 3:** Difference-in-difference Poisson regression models estimating the effects of SNAP issuance periods on SSB ad counts, adjusting for state and calendar time

	Short issuance (CT, 3 d; NE and NJ, 5 d)		Long issuance (CA, 10 d; TX, 15 d)		All states combined	
	Mean	sd	Mean	sd	Mean	sd
Summary statistics (all stores)						
% Florida SSB ads in circulars during comparison states’ issuance days, mean (sd)	46·7	13·4	47·2	13·9	47·2	13·9
% Florida SSB ads in circulars during comparison states’ non-issuance days, mean (sd)	48·2	12·7	48·5	11·6	48·5	11·6
% Comparison states’ SSB ads in circulars during issuance days, mean (sd)	50·0	15·9	52·4	18·3	51·5	17·5
% Comparison states’ SSB ads in circulars during non-issuance days, mean (sd)	47·2	15·5	50·7	19·0	48·3	16·7
	b	95 % CI	b	95 % CI	b	95 % CI
Coefficient estimates						
**State** effect: Relative difference from FL, b (95 % CI)^*^	0·987	0·945, 1·031	1·032	0·978, 1·088	1·002	0·961, 1·045
**State** effect: Adjusted absolute difference from FL, b (95 % CI)^§^	**−0·143**	**–0·629, 0·343**	**0·260**	**–0·183, 0·702**	0·019	–0·401, 0·439
**Time** effect: Issuance relative difference, b (95 % CI)^†^	**0·983**	**0·976, 0·991**	**0·985**	**0·977, 0·993**	**0·984**	**0·976, 0·993**
**Time** effect: Absolute difference from issuance period, b (95 % CI)^§^	−0·186	–0·274, –0·099	**−0·129**	**–0·197, –0·061**	**−0·160**	**–0·245, –0·076**
**State × Time** effect: Relative difference in differences, b (95 % CI)^‡^	**1·031**	**1·022, 1·040**	**1·025**	**1·011, 1·039**	**1·029**	**1·019, 1·039**
**State × Time** effect: Absolute difference in differences, b (95 % CI)^§^	**0·339**	**0·239, 0·440**	**0·206**	**0·095, 0·317**	**0·284**	**0·187, 0·381**
Observations	24 171		18 004		36 064	

SNAP, Supplemental Nutrition Assistance Program; SSB, sugar-sweetened beverages.

Author’s analysis of daily ads derived from the weekly circulars of 563 stores in six states collected from August to September 2019. For each issuance duration in each column, the issuance period for Florida was reassigned to coincide with the longest issuance period of the comparison states before estimation. For example, when compared to short-issuance states (CT, NE and NJ), the issuance period in FL was recoded to occur in the first 5 d of the month. When compared to CA and TX, and to all states, the issuance days in FL were recoded to occur in the first 15 d of the month. All difference estimates are derived from Poisson difference-in-difference regressions, and they indicate the effect of issuance, state and their interaction on the number of SSB ads. Total number of beverage ads is included in each regression as a covariate with its coefficient constrained to 1. All models include state-level fixed effects, store location- and week-level random effects. Robust standard errors are adjusted for clustering within store.

Boldface indicates statistically significant difference at *P* < 0·05.

* From the coefficient on the indicator variable for state that is equal to one for the comparison state or states and zero for Florida.

† From the coefficient on the indicator variable for SNAP issuance day.

‡ From the coefficient on the interaction of SNAP issuance day and the indicator for the comparison state or states.

§ From the margins command in Stata, version 15.

## Discussion

This study was the first, to our knowledge, to leverage longitudinal data from a sample of SNAP-authorised retailers across the USA to estimate the association between SNAP issuance timing and SSB marketing. SSB accounted for the highest proportion of beverage ads in weekly circulars, regardless of the time of month or the state; SSB were 48·4 % of non-issuance beverage ads and 51·5 % of in-issuance beverage ads. Further, healthier beverage ads (e.g. unsweetened) were less prevalent when circulars were in-issuance compared to non-issuance. Using a cross-state, difference-in-differences analysis, we also found that the proportion of beverage ads for SSB increased slightly – by about 3 % – during weeks when SNAP benefits were being issued compared to when benefits were not in issuance.

While this study did find a small increase in the prevalence of SSB advertisements in circulars associated with SNAP benefit issuance periods, the difference was not as large as what has been identified a prior study by Moran *et al.* comparing in-store marketing during issuance periods and non-issuance period in New York state^(31)^. This prior study of in-store marketing found a substantially higher likelihood of such SSB marketing during SNAP issuance periods compared to non-issuance periods (i.e. a 66–88 % increase in the odds of an in-store SSB ad being present), which was even more pronounced in retailers located in neighbourhoods with a high proportion of SNAP participants. The substantially smaller magnitude of the association found in this current study may be due to several key differences. By not adjusting for time trends, the prior study may have only captured the ‘first of the month effect’, whereby SNAP participants do more shopping at the start of the month^(42)^. It also studied different marketing strategies: whereas this study examined circulars from the store website, the prior study assessed in-store signage, ads and displays via direct observation. In-store marketing can be localised and flexible, and up to the discretion of a given store manager, while circulars may be developed far in advance of circulation, thus being less flexible to time-sensitive promotions. And, for franchised supermarket chains that cross multiple states, circulars may also be developed at a franchise level and thus have less localised strategies. Finally, there may have been unobserved attributes unique to upstate New York that contributed to disproportionate marketing at the beginning of the month.

Strengths of this study include the large sample size and the random sampling scheme, which helped ensure adequate power and that the circulars in our sample were likely representative of circulars for SNAP-authorised retailers. Our selection methodology of states where the most SNAP benefits were redeemed in each issuance group (short, long and continuous) is also a strength, as SNAP-authorised retailers may be most incentivised to match circulars marketing strategies to issuance timing.

A key limitation is that we are not able to assess whether the observed association between SSB marketing and SNAP benefit issuance timing leads to changes in SSB purchasing behaviour for SNAP households. While this study was able to account for possible time trends in SSB ads by comparing the differences in ads between benefit issuance and non-issuance periods with the same time periods in a state that was continuously issuing benefits, it cannot rule out that the observed small uptick in SSB ads during SNAP benefit issuance periods is not due to some other factor besides SNAP benefit issuance. For example, while FL was the most appropriate state in our available data to use as a control because of its continuous issuance period, there may have been unique, unobserved market dynamics in FL that explain why it was statistically significantly different from the other states in our sample (Table 1). Another limitation is that because we excluded stores with at least 1 week of missing circulars and were unable to sample from stores without digital circulars, there remains uncertainty in our estimates. Finally, because SNAP participants may have less Internet access than SNAP non-participants^(28,29)^, it is possible that greater exposure to SSB marketing will be less relevant for SNAP participants.

Circulars raise consumers’ awareness of in-store promotions, which can increase the purchasing of promoted items^(23,26,35)^. Although the number of SSB ads in circulars was only slightly higher during SNAP benefit issuance periods, it is possible that sustained exposure over time may contribute to increased SSB purchasing behaviour during SNAP issuance, particularly for households participating in SNAP, especially considering most benefits are spent during benefit issuance periods. Additionally, we observed that SSB ads constituted the majority of beverage ads in circulars regardless of SNAP issuance timing. Thus, these beverages are heavily marketed to all consumers – this is consistent with data on circulars marketing that unhealthier beverage and food products are frequently promoted^(24,40,43,44)^.

In conclusion, our findings suggest that, at any point in the SNAP issuance cycle, consumers are exposed to a very high volume of SSB ads through weekly circulars. In one week, a circular can be expected to promote an average of between six and sixteen SSB products, which outpaces promotions for any other beverage type. Diet and low-calorie beverages, for example, are featured less than twice in an average circular in any state. In addition, the relative volume of SSB ads is slightly higher during issuance compared to non-issuance periods, but in general, one in two beverages advertised are SSB. Future research should further explore the complex linkages between marketing strategies and consumer behaviour related to SSB purchasing and consumption, such as how knowledge about SNAP benefit issuance timing is applied to both in-store and circulars marketing strategies as well as how and which SNAP recipients use circulars for their purchasing decisions.

## Supporting information

Dai et al. supplementary material Dai et al. supplementary material

## Supplementary material

For supplementary material accompanying this paper visit https://doi.org/10.1017/S1368980024001563
