# Supplementary material for: Supplemental Nutrition Assistance Program issuance timing is associated with sugar-sweetened beverage marketing in the USA
Source: Public Health Nutr. 2024 Sep 23;27(1):e167. doi: 10.1017/S1368980024001563 (PMC11504265; doi:10.1017/S1368980024001563)
Supplement: Dai et al. supplementary material [file S1368980024001563sup001.docx]

# **Supplemental Information.** Data extraction protocol.

*Online data collection and management: Box, Google Drive*

## Circular Assignments

1. All circulars for extraction can be found in Box. If you don’t have access to the files, email XX and XX will get you set up.
2. The circulars you are assigned to extract will be found in the Google Sheet under your name. Store details are pre-populated, so all you need to do is add the extracted data.
   1. Circulars will be organized by store when they are assigned. However, we encourage you to re-sort as needed (e.g, by date of issuance). This is a good idea when stores issue the same circular across multiple locations.

## Ad Categories

Group discussions have led us to define all ads among 5 groups—Beverages, Other (includes Food, Alcohol, Non-edibles), and Mixed (includes Beverages and other items, usually food, in the same ad). We want to understand the proportion of ads that are in each category compared to the total number of ads.

**Scheme 1: Decision tree for classifying ads**

Follow this chart to categorize ads—for additional help in discerning pictures and texts in the ad, refer to **Scheme 2** (picture classification) and/or **Scheme 3** (Text classification).

1. For each circular, count how many ads are in each category. Sum up and enter into Google Sheet.

- An ad must be itemized: has a product name and a corresponding price. Not all ads will have photos (e.g., Publix)

1. [Beverage Study] For ads with beverages *and* other items (e.g., Coke and chips), we still want to extract information about the beverages since they are being promoted in the circular. Include these as Mixed ads.

Scheme 1. Decision tree for classifying ads

Scheme 2. Picture classification decision tree for classifying ads

Scheme 3. Text classification decision tree for classifying ads

## Beverage extraction

For this first study, we want to know how many beverages fall into 4 categories: water/unsweetened, diet/low-cal, SSB, and milk/100% juice.

1. Identify the type of beverage ad – the dominant beverage is what defines the ad. See **Schemes 2 and 3** for the relevant decision trees.
2. Follow the extraction protocol for all beverage ads.
3. Follow the extraction protocol for all Mixed ads that include beverages. If the mixed ad includes a single beverage, consider it the dominant beverage for data extraction. If there are several beverages in the Mixed ad, follow the same decision tree (item that is most dominant in the image, or dominant in the text, if relevant), to identify as the dominant beverage item in the Mixed ad. Extract data as available.
4. Sum up counts for all beverage subtypes and add to Google Sheet. There are two separate “sums”, one for ads that were Beverage-only ads, and one for ads that were Mixed ads that featured beverages.
5. Sum up all “Other” ads. This enables us to calculate a proportion of SSB ads from the entire circular.

## End of survey – checks

1. Add up the total number of beverage and Mixed ads (Beverage Study) and beverage and food ads (Processing Study) and enter into Google Sheet. There are automated columns that will serve as checks to make sure the numbers add up properly.
2. **Mark in Google Sheet the date of extraction.** Let XX know when you’ve completed your assignment, and then XX will update your Sheet with the next batch of circulars.
